# Level of Late Initiation of Antenatal Care Visit and Associated Factors Amongst Antenatal Care Attendant Mothers in Gedo General Hospital, West Shoa Zone, Oromia Region, Ethiopia

**DOI:** 10.3389/fpubh.2022.866030

**Published:** 2022-06-20

**Authors:** Bikila Tefera Debelo, Kababa Temesgen Danusa

**Affiliations:** Department of Midwifery, College of Medicine and Health Sciences, Ambo University, Ambo, Ethiopia

**Keywords:** late initiation, antenatal care, pregnant, women, Gedo Hospital, Ethiopia

## Abstract

**Background:**

Antenatal care is a care given for pregnant women and is taken as a key maternal care service in improving and keeping health of both life outcomes for mothers and newborns. Countries with low antenatal care coverage are the countries with very high maternal mortality ratios.

**Objective:**

Hence, The aim of this study was to determine the level of late initiation of antenatal care visit and associated factors amongst antenatal care follow up in Antenatal care (ANC) services at Gedo General Hospital, Western Oromia Region, Ethiopia, 2021

**Methodology:**

A health facility based cross-sectional study design was conducted from July 10–30, 2021 using primary data review and face-to-face interviews among clients receiving ANC. A total of 347 mothers was selected by simple random sampling and were interviewed while they come to antenatal care follow-up in Gedo general hospital. Data were entered into Epi-data version 4.6 and then changed to SPSS version 23 for the analysis purpose. Those Variables which are *P* < 0.25 in binary logistic regression were selected as a candidate for multiple logistic regressions to determine independently associated factors. The adjusted odds ratio was employed with 95% CI to illustrate the strength of association and *P* < 0.05 was used to state a statistical significance.

**Result:**

Among 330 women, about 58.5% of women came for their first ANC visit initiation lately. Being a housewife, having a family size >4, and having a distance from the health facility >1 h were higher odds of late first ANC visit initiation as compared to *vice versa*. Besides, women aged 20–24 years had 0.18 times and 25–29 years had lower odds of late first ANC visit initiation

**Conclusion:**

Majority of women began their first antenatal care initiation lately. Therefore, the provision of awareness on the significance of attending the first antenatal care early *via* health extension workers is recommended

## Introduction

Antenatal care is the care offered for pregnant women and is recognized as a input for maternal service in improving and keeping health outcomes for both lives of mothers and newborns. World health organization (WHO) ANC model recommends a minimum of four ANC contacts, and encourages that the first contact appointment will take place within the first trimester (1). Antenatal care is also referred as the care provided by skilled healthcare providers to pregnant mothers to make sure the health conditions for both mother and baby. ANC is one of the “four pillars” of safe motherhood initiatives, which is used to promote and establish good health during pregnancy and the early postpartum periods (2–4).

Globally, there is a huge difference in the prevalence of late ANC checkup among pregnant mothers that is the prevalence of late ANC in developed countries is 27.5%, and that of developing countries is 88 % (5–8). In spite of ANC service is given without fee and there is high accessibility; low ANC utilization and late registering or booking is still a main problem (9, 10).

Even though, most studies have revealed a lot of factors affecting the utilization of ANC in different contexts, these findings have not been prepared collectively for a factor that affects mothers for late ANC initiation (11). According to a study conducted in Mekelle city, Most of the attendees 109 (48%) made their first visit in their first trimesters and a study done in Jimma town about (51.8%) of the mothers started ANC at late (12–14).

Women who have an inadequate number of ANC visits or start ANC later than the first trimester seem to have higher rates of poor pregnancy outcomes, such as low birth weight and pre-term birth (15, 16). Maternal health complications and lack of prenatal outcomes are often associated with other factors like the non-utilization of antenatal and childbirth services and poor socioeconomic status. Unwanted outcomes are, seen in those mothers who have not done ANC registration due to their delay of antenatal care because of dissatisfaction toward the quality of service pregnant mothers are affected by complications like: preeclampsia, malaria, lack of birth readiness, post-partum hemorrhage due to lack of risk identification at ANC level (17–23).

According to a study done in Niger Delta, Nigeria 73.6% of mothers booked lately (24). According to research conducted in Douala general hospital (DGH), Cameroon From a total of participated women, about 44% of them registered the first ANC visit lately (25). A study done in public health institutions of Axum town, the prevalence of timely ANC initiation of was 27.5%. But, about three fourth of the mothers (72.5%) booked their ANC visit lately (26). A study conducted in Kembata Tembaro Zone in SNNPR also shows that the magnitude of participants who started ANC early was 31.4% while those who registered lately were 68.6%. The finding of the study conducted in northern Ethiopia shows that 59.4% of pregnant mothers began their first visit after the first trimester and in the central zone of the Tigray region the prevalence of late ANC booking at the first visit was 85% (1, 8, 19, 27–29).

According to a study done in Debre Markos town, the magnitude of participants who made their first antenatal care visit after 16 weeks of gestation was found to be 33% (30). A done in Debre Berhan town, the proportion of pregnant women who had ANC booking within the first trimester of pregnancy was 40.6% (31). According to a study done in Ilu Ababor Oromia regional state, the proportion of respondents who initiated ANC lately was 71.2% (32).

There is no study done on the time of ANC initiation amongst pregnant mothers in the selected study area to the best of our knowledge. It is essential to identify the status of ANC late initiation and associated factors to improve maternal and fetal outcomes via sufficient utilization of Antenatal care.

## Materials and Methods

### Study Setting, Design, Population

The study was conducted in Gedo General Hospital in west Shoa of Oromia regional state west of Ethiopia, which is about 178 kilometers from the capital city, Addis Ababa, and 64 km from Ambo town.it is located at the east of Gedo town 3 km away from the bus station. Gedo General Hospital is a General Hospital owned by the Government. It has four major clinical departments (medical and surgical, pediatrics and Gynecology or Obstetrics) and other minor departments psychiatry, Nice, TB, along with other follow-up and special clinics for specific diseases such as Antiretroviral therapy (ART) and Tuberclosisis (TB). This study was, conducted from May, 15/2021 up to June 15/2021. An institutional-based cross-sectional study design was employed. The source of the population of this study was all pregnant women came for ANC follow-up at Gedo General Hospital and the study population was selected pregnant mothers visiting ANC in Gedo General Hospital during the study period and fulfilling the eligibility criteria. The inclusion criteria for the study were selected pregnant mothers visiting ANC service in Gedo General Hospital during the data collection period.

### Sample Size Determination and Sampling Technique

The sample size was determined using a single population proportion formula by considering the proportion of ANC late initiation, which is 71.2% in Ilu Ababora Zone, southwest Ethiopia, 95% significance level of Z α/2 = 1.96, 5% margin of error, and 10% of non-response rate (33).


$$\begin{array}{l} n=\frac{{\left(Z\alpha /2\right)}^{2}xP\left(1-P\right)}{d^{2}}=n=315 \end{array}$$

Hence, the total sample size = 315 + 10 % of none response rate = 347.

A probability systematic random sampling technique was employed by using constant K value (K = *N*/*n*, 600/347 = 1.7 ≈ 2), *N* is the total number of ANC attendants in the last year's quarterly case flow report of the same periods (*N* = 600). Every two client was interviewed by using a systematic sampling technique. The first client to be interviewed was selected randomly by lottery method from the first two client. The exit interview technique was used to collect data on their clinic days.

### Data Collection Tool and Techniques

The data was collected using a pretested structured questionnaire administered face-to-face interview. Pregnant women's knowledge on the advantage and services availability of ANC was asked using 10 questions. The questionnaire was developed from different pieces of literature (33). It was prepared in English and translated to Afan Oromo (local language) for clarification and back to English for data analysis and to check its consistency. The local language version of the questionnaire was used to collect the data. There are four Midwifery student data collectors with supervision turned by the turn supervisor of the investigator who can be closely supervising the process of data collection.

### Variables and Operational Definition

#### Dependent Variable: Late Initiation of ANC (Yes, No)

Independent variables were socio-demographic variables: Maternal Age, Religion, marital status, ethnicity, residence, income, occupation, employment family size, and educational level. Obstetric factors: Gravidity, Parity, GA, Birth interval, previous and current ANC use, number of ANC visits, Maternal medical conditions (DM and Hypertension), Previous mode of delivery, previous CS scar, number of previous CS done. Maternal behavior: chat chewing, drinking alcohol, cigarette smoking.

#### Data Collection Method and Procedure

*Late initiation of antenatal care*: Refers to a woman who came for ANC beyond 16 weeks gestation for the first time during the pregnancy (26). *Antenatal care* is a type of care given for pregnant women and is considered a key maternal service in improving a wide range of health outcomes for women and newborns (29).

#### Data Processing and Analysis

The collected data was cleaned, edited, coded, and entered into Epi-data V- 4.6 and exported to SPSS version V-23.0 for analysis. After categorizing and defining variables, a descriptive analysis will be employed for each of the independent variables. The results were reported using percentages, tables, and graphs. Bivariate analysis variables that indicate significant association with 95% CI and a *P* < 0.25 were entered in to multivariable analysis to select the factors associated with the ANC late initiation. The screened variables were fitted to the multivariable logistic regression model through a backward stepwise method to reduce the effects of confounders. Adjusted odds ratio (AOR) was calculated with 95% CI to show the strength of association and *P* < 0.05 was used to assert statistical significance.

#### Data Quality Control

The data collected was cleared and checked for completeness of data. The quality of the questionnaire will be secured by pre-testing the tool and training the interviewers and supervisors previous to the real data collection. The pre-test of the questionnaire, which is 5% of the total sample size, was carried out in Ambo health facility- outside of the selected study area. Training was given to interviewers and supervisors on the data collection techniques. The completeness, clarity and consistency of the data collected will be checked by supervisors and investigators.

### Ethics Statement

Before the data collection procedure, an ethical clearance letter was taken from the Ambo University department of midwifery ethical committee with the reference number UGC/83/2021, and similarly, permission from Gedo General Hospital was obtained. Oral and written consent was obtained from the responder. The respondents' rights and dignity were also respected. Confidentiality was, kept throughout data collection and the woman has a right to refuse or discontinue any time during the interview. The interview was undertaken in a private area to keep privacy and confidentiality.

## Results

### Socio-Demographic Characteristics

In this study, 330 pregnant women participated, with a response rate of 95%. Among all respondents about 115 (34.8%) pregnant women were found between the age of 20–24 years. The majority 224 (67.9%) of mothers were protestant by religion. About two-thirds, 309 (93.6%) were married and most of them 228 (69.1%) are literate in educational status. The majority of the respondents were housewives which accounted for 234 (70.9%) (Table 1).

**Table 1:** Socio-demographic characteristics of pregnant mothers who were attending ANC in the Gedo general Hospital, West Shoa zone, Oromia region, Ethiopia (*n* = 330).

**Variables**	**Categories**	**Early ANC initiation**	**Late ANC initiation**	**Total**
		***N*** **(%)**	***N*** **(%)**	***N*** **(%)**
Age	15–19	4 (2.9)	18 (9.3%)	22 (6.7)
	20–24	57 (41.6%)	58 (30.1%)	115 (34.8)
	25–29	24 (17.5%)	41 (21.2%)	65 (19.7)
	30–34	40 (29.2%)	66 (34.2%)	106 (32.1)
	35–39	12 (8.8%)	10 (5.2%)	22 (6.7)
	Total	137 (100)	193 (100)	330 (100)
Religion	Orthodox	39 (28.5)	51 (26.4)	90 (27.3)
	Protestant	86 (62.8)	138 (71.5)	224 (67.9)
	Muslim	10 (7.3)	4 (2.1)	14 (4.2)
	Others	2 (1.5)	0	2 (0.5)
	Total	137 (100)	193 (100)	330 (100)
Marital status	Single	8 (5.8)	9 (4.7%)	17 (5.2)
	Married	127 (92)	182 (94)	309 (93.6)
	Divorced/widowed	2 (1.5)	2 (1)	4 (1.2)
	Total	137 (100)	193 (100)	330 (100)
Residence	Urban	61 (44.5)	53 (27.5)	114 (34.5)
	Rural	76 (55.5)	140 (72.5)	216 (65.5)
	Total	137 (100)	193 (100)	330 (100)
Educational status	Illiterate	68 (49.6)	34 (17.6)	102 (30.9)
	Literate	69 (50.4)	159 (82.4)	228 (69.1)
	Total	137 (100)	193 (100)	330 (100)
Maternal occupation	Government employee	48 (35)	48 (24.9)	96 (29.1)
	Housewife	89 (65)	145 (75)	234 (70.9)
	Total	137 (100)	193 (100)	330 (100)
Husband occupation	government employee	16 (12.7)	23 (12.6)	40 (12.6)
	Merchant	28 (22.20)	59 (32.1)	87 (28.1)
	Farmer	729 (57.1)	65 (35.3)	137 (44.2)
	Student/daily labourer	10 (7.9)	36 (19.6)	46 (14.8)
	Total	126 (100)	183 (100)	309 (100)
Estimated monthly income	<1,000	10 (7.3)	6 (3.1)	16 (4.8)
	1,000–2,987.50	103 (75.2)	156 (80.8)	259 (78.5)
	>2,987.50	24 (17.5)	31 (16.1)	55 (16.7)
	Total	137 (100)	193 (100)	330 (100)
Family size	≤ 3	64 (46.7)	139 (72)	203 (61.5)
	≥4	73 (53.3)	54 (28)	127 (38.5)
	Total	137 (100)	193 (100)	330 (100)
Distance	<1 h	72 (52.6)	30 (15.5)	102 (30.9)
	≥1 h	65 (47.4)	163 (84.5)	228 (69.1)
	Total	137 (100)	193 (100)	330 (100)

### Time of First Antenatal Care Visit

**Figure 1:**
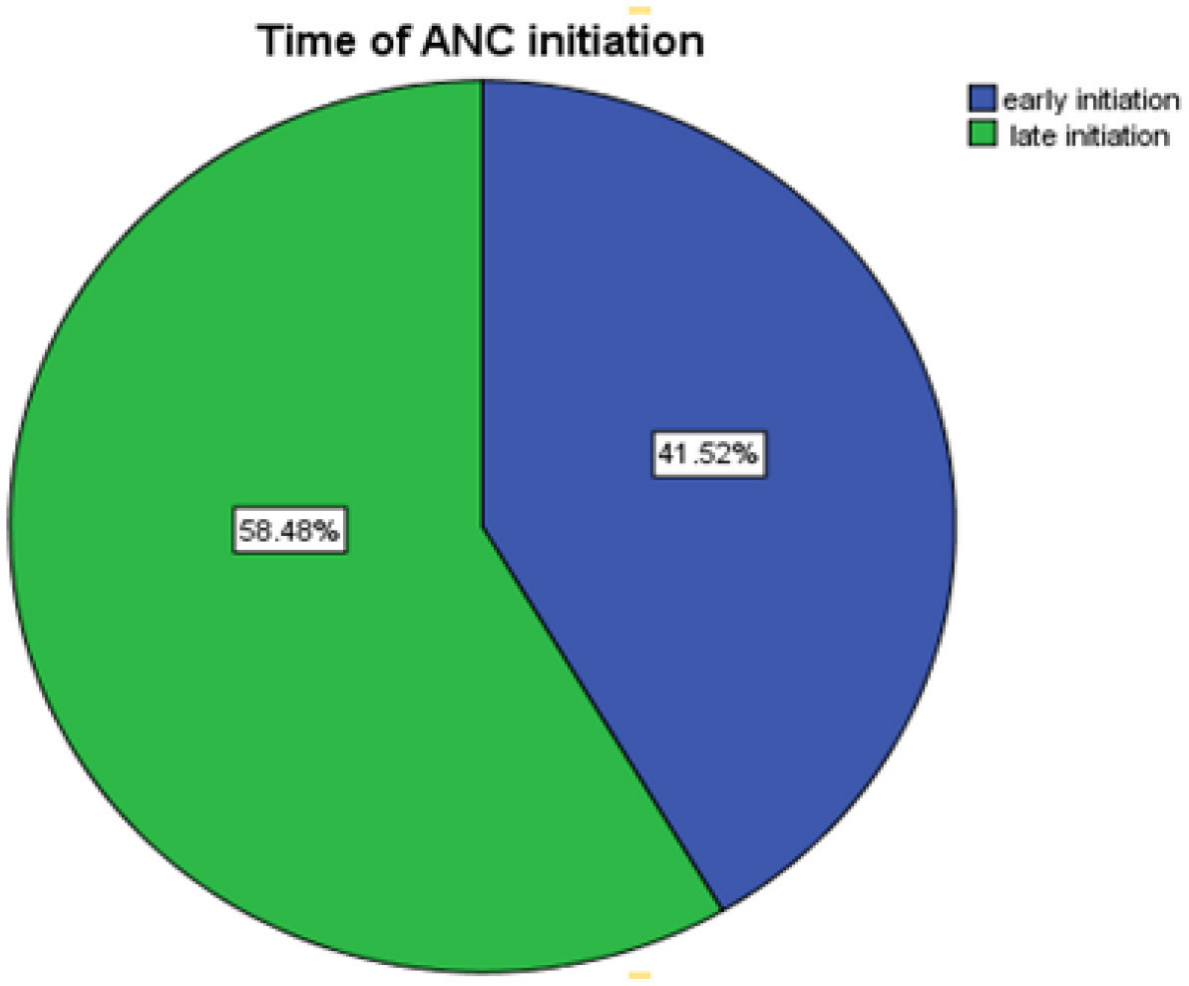
Time the first antenatal care visit initiation who were attending ANC in Gedo General Hospital (*n* = 330).

The proportion of respondents who initiated their first ANC lately was 193 (58.5%) and early first ANC visit initiation was 137 (48.5%) (Figure 1). Moreover, most of the women 155 (47%) used 3rd ANC visit and 128 (38.8%) of them decided with their husband to initiate ANC visit. The majority of the 219 (66.4%) did not Inform when to register 1st ANC and 201 (60.9) did not use ambulance services. The majority of the participants 279 (84.5%) have awareness regarding danger signs and 316 (95.8%) have usage of ANC for birth preparedness (Table 2).

**Table 2:** Obstetric history of pregnant mothers, who were attending ANC in Gedo general hospital (*n* = 330).

**Variables**	**Category**	**Initiation of ANC**		**Total number (%)**
		**Early number (%)**	**Lately number (%)**	
Gravidity	Primigravida	15 (10.9)	26 (13.5)	41 (12.4)
	Multigravida	122 (89.1)	167 (66.5)	289 (87.6)
Parity	Para zero	13 (9.5)	24 (12.4)	37 (11.2)
	Para one and above	124 (90.5)	169 (87.6)	293 (88.8)
Abortion	Yes	12 (8.8)	20 (10.4)	32 (9.7)
	No	125 (91.2)	173 (89.6)	298 (90.3)
History of preterm	Yes	9 (6.6)	6 (3.1)	15 (4.5)
	No	128 (93.4)	187 (96.9)	315 (95.5)
Number of ANC visit	One	10 (7.3)	29 (15)	39 (11.8)
	Two	57 (41.6)	98 (50.8)	155 (47)
	Three	49 (35.8)	52 (26.9)	101 (30.6)
	Four and above	21 (15.3)	14 (7.3)	35 (10.6)
who decided to initiate ANC	Self	43 (31.4)	66 (34.2)	109 (33)
	Husband	15 (10.9)	16 (8.3)	31 (9.4)
	family members	30 (21.9)	32 (16.6)	62 (18.8)
	wife and husband	49 (35.8)	79 (40.9)	128 (38.8)
Informed when to register 1st ANC	Yes	73 (53.3)	38 (19.7)	111 (33.6)
	No	64 (46.7)	155 (800)	219 (66.4)
Use of ambulance	Yes	31 (22.6)	98 (50.8)	129 (39.1)
	No	106 (77)	95 (49)	201 (60.9)
Danger sign awareness	Yes	110 (80.3)	169 (87.6)	279 (84.5)
	No	27 (19.7)	24 (12.4)	51 (15.5)
Does ANC use birth preparedness	Yes	125991.2	191 (99)	316 (95.8)
	No	12 (8.8)	2 (1)	14 (4.2)

### Obstetric History of the Respondents

Two hundred eighty-nine (87.6%) of the respondents were multigravida. Most 293 (88.8%) of the women were part one and above. Among the total respondents, 32 (9.2%) had a history of abortion (Table 2).

### Factors Associated With the Late First ANC Visit

On bivariate analysis, factors found to be significantly associated with the late initiation of ANC visit was age, residence, maternal occupation, estimated monthly income, family size, gravidity, distance to the health facility, informed when to register the first ANC visit, Ambulance service, number of ANC visit, awareness of danger sign, awareness of ANC usage for birth preparedness having a history of preterm, On multivariable analysis, age, maternal occupation, family size, distance to the health facility, Ambulance service, were significantly associated with the late booking of the first ANC visit.

The odds of late initiation of ANC for women aged between 20 and 24 years had 0.18 times (AOR: 0.18; 95% CI: 0.05, 0.63) and 25–29 years had 0.24 times (AOR: 0.24; 95% CI: 0.06, 0.90) the odds of those women aged between 15 and 19 years. The odds of late initiation of ANC for women who were housewives in their occupation had 2.22 times (AOR: 2.22; 95% CI: 1.17, 4.19) the odds of those women who were governmental employees. The odds of late initiation of ANC for women who were living within a family size of greater than and equal to four is 2.29 times (AOR: 2.29; 95% CI: 1.24, 4.22) the odds of those living within less and equal to three family size. The odds of late initiation of ANC for women who travel >1 h and above of walking time from their home to health facility had 5.52 times (AOR: 5.52; 95% CI: 2.71, 11.22) the odds of those who travel <1 h and above of walking time. The odds of late initiation of ANC for women who got Ambulance service had 0.38 times (AOR: 0.38; 95% CI: 0.21, 0.68) the odds of those women who did not get Ambulance service (Table 3).

**Table 3:** Multivariable model of the factors for late ANC initiation among pregnant women attending Gedo Primary Hospital, West Shoa Zone, Oromia region, Ethiopia, 2021.

**Variables**	**Categories**	**Early ANC initiation *N* (%)**	**Late ANC initiation *N* (%)**	**COR**	**AOR**	***P*-value**
Age	15–19	4 (2.9)	18 (9.3%	1	1	
	20–24	57 (41.6%	58 (30.1%	0.22 (0.07, 0.71)	**0.18 (0.05, 0.63)**	**0.008**
	25–29	24 (17.5%	41 (21.2%	0.38 (0.11, 1.25)	**0.24 (0.06, 0.90)**	**0.035**
	30–34	40 (29.2%	66 (34.2%	0.36 (0.11, 1.16)	0.32 (0.08, 1.16)	0.084
	35–39	12 (8.8%	10 (5.2%	0.18 (0.04, 0.73)	0.37 (0.07, 1.90)	0.238
Residence	Urban	61 (44.5)	53 (27.5)	1	1	
	Rural	76 (55.5)	140 (72.5)	2.12 (1.33, 3.36)	0.54 (0.27, 1.09)	0.08
Educational status	Illiterate	68 (49.6)	34 (17.6)	1	1	
	Literate	69 (50.4)	159 (82.4)	4.6 (2.79, 7.59)	0.34 (0.62, 1.86)	0.21
Maternal occupation	Government employee	48 (35)	48 (24.9)	1	1	
	Housewife	89 (65)	145 (75)	1.62 (1.01, 2.63)	2.22 (1.17, 4.19)	0.01
Estimated monthly income	<1,000	10 (7.3)	6 (3.1)	1	1	
	1,000–2,987.50	103 (75.2)	156 (80.8)	2.524 (0.89, 7.15)	2.39 (0.62, 9.18)	0.20
	>2,987.50	24 (17.5)	31 (16.1)	2.153 (0.68, 6.75)	1.07 (0.25, 4.58)	0.92
Family size	≤ 3	64 (46.7)	139 (72)	1	1	
	≥4	73 (53.3)	54 (28)	2.93 (1.85, 4.65)	**2.29 (1.24, 4.22)**	**0.008**
Distance	<1 h	72 (52.6)	30 (15.5)	1	1	
	≥1 h	65 (47.4)	163 (84.5)	6.01 (3.6, 10.06)	**5.52 (2.71, 11.22)**	**0.001**
Informed when to book 1^st^ ANC	Yes	73 (53.3)	38 (19.7)	1	1	
	No	64 (46.7)	155 (800)	4.65 (2.85, 7.58)	1.57 (0.55, 4.52)	0.395
Use of Ambulance	Yes	31 (22.6)	98 (50.8)	1	1	
	No	106 (77)	95 (49)	0.28 (0.17, 0.46)	**0.38 (0.21, 0.68)**	**0.001**
No of ANC visit	One	10 (7.3)	29 (15)	1	1	
	Two	57 (41.6)	98 (50.8)	0.59 (0.26, 0.30)	0.79 (0.29, 2.13)	0.65
	Three	49 (35.8)	52 (26.9)	0.36 (0.16, 0.83)	0.42 (0.15, 1.18)	0.10
	Four and above	21 (15.3)	14 (7.3)	0.23 (0.08, 0.61)	0.33 (0.09, 1.13)	0.07
Danger sign awareness	Yes	110 (80.3)	169 (87.6)	1	1	
	No	27 (19.7)	24 (12.4)	0.579 (0.318, 0.054)	0.64 (0.26, 1.58)	0.33
Does ANC use birth preparedness	Yes	125991.2	191 (99)	1	1	
	No	12 (8.8)	2 (1)	0.57 (0.318, 1.05)	0.24 (0.04, 1.28)	0.09
History of preterm	Yes	9 (6.6)	6 (3.1)	1	1	
	No	128 (93.4)	187 (96.9)	0.57 (0.31, 1.05)	1.81 (0.49, 6.60)	0.36

*Bold values indicate significant association at a P-value ≤ 0.05*.

## Discussion

The results of this study showed that among 330 women, about 58.5 % of women came for their first ANC visit initiation lately or registered first ANC visit after 16 weeks of gestation. The finding of this study is almost higher than when compared to with a study done in Douala general hospital (DGH), Cameroon 44 % (25), Debre Markos, 33.4 % (30). This is might be due to socio-demographic differences and health care service awareness. But the prevalence of women who started for their first ANC visit lately is significantly lower than Nigeria 73.6% (24), Axum town (72.5%) (26), Kembata Tembaro Zone (68.6%) (27), Ilu Ababor (71.2%) (33), East Wollega 81.5% (23). This is because of the high distribution of health posts and the promotion of maternal health care utilization by health extension workers.

Furthermore, the magnitude of respondents who registered lately in this study is lower than the Ethiopian demographic health survey 2019 report of 72% or only 28% of women had their first ANC visit during the first trimester (34). This may be due to this study being at a specific hospital in which total populations are different when we compare with the national and gap in the year of study and a lot of strategies and work has been done since then to improve maternal health services.

In this study, teenagers mothers were utilized late ANC than older women. This finding is similar with the studies done in Zambia (35), Nigeria (36), and the United Kingdom (37). This might be young teenagers women may have lack of knowledge, single, unwanted pregnancy, did not experienced with pregnancy when compared with women aged >20 years (35–37). Women who were housewives in their occupation were came lately to ANC visit as compared to those mothers who were governmental employees. This finding was similar with other studies done in North West of Ethiopia (38). The probable justification might be that those women who were governmental employee might have better access of knowledge, peer pressure, awareness and understanding about early ANC services (39).

Mothers who were living with high family size were came lately ANC visit initiation as compared to those who were living with less and equal to three family size. This study is in line with the study done in Rwanda (40) and Illu Abbabora, Ethiopia (33). This might be due to lack of economy which increases as the number of family size increases in developing country. Another reason might be most women overtake their time on other workload activities to develop their family to fulfill their basic needs rather than considering about their pregnancy checkup early.

Regarding distance, Women who travel >1 h and above of walking time from their home to the health facility had 5.52 times higher odds of late first ANC visit initiation as compared to those who travel <1 h and above of walking time. This study is in line with a study done in Zambia, Bahirdar Zuria Wereda, Ethiopia (35, 41). The possible reason might be those women living in a low income country utilize their time in day to day activity for survival of them self's and their family rather than considering their health checkup until they feel some symptoms. The other reason might be due to lack of transport and uncomfortable road for vehicles due to most valley land or unsuitable geographical area.

### Limitations of the Study

First, the nature of this study design is subject to selection bias and measurement bias. Women who were at private clinic were not included which led to a lack of generalization was recognized as a limitation of the study.

## Conclusions

In this study, the majority of women started their first antenatal care initiation lately. Women's age, being housewives, having a large family size, and long-distance from a health facility were factors identified for late first ANC initiation. Based on the findings of this study, Ambo Zonal Health Office and Cheliya Wereda Health Office should strengthen the provision of training for health extension workers to teach pregnant mothers about early ANC initiation by going home to home. Provision of education and communication for young mothers and their partners by health professionals at a hospital during morning health education and Provision of additional Hospital or ambulance for the community to reach easily and timely at health facility service.

## Data Availability Statement

The original contributions presented in the study are included in the article/supplementary material, further inquiries can be directed to the corresponding authors.

## Ethics Statement

An ethical clearance letter was obtained from the Ambo University Department of Midwifery Ethical Committee with the reference number UGC/83/2021. Written informed consent to participate in this study was provided by the participants' legal guardian/next of kin.

## Author Contributions

KD and BD conceptualized the idea, wrote the original draft, supervised the overall study, analyzed data, prepared the manuscript, drafted or revised the article, gave final approval of the version to be published, and agree to be accountable for all aspects of the work.

## Conflict of Interest

The authors declare that the research was conducted in the absence of any commercial or financial relationships that could be construed as a potential conflict of interest.

## Publisher's Note

All claims expressed in this article are solely those of the authors and do not necessarily represent those of their affiliated organizations, or those of the publisher, the editors and the reviewers. Any product that may be evaluated in this article, or claim that may be made by its manufacturer, is not guaranteed or endorsed by the publisher.

## Abbreviations

ANC: Antenatal Care
AOR: Adjusted Odd Ratio
CI: Confidence Interval
EDHS: Ethiopian Demographic and Health Survey
FANC: Focused Antenatal care
IMR: Infant Mortality Rate
MMR: Maternal Mortality Rate
WHO: World Health Organization.
